# 

**Published:** 1842-10-15

**Authors:** 


**CONTENTS OF No. 42.**

Wilson's Cases of Puerperal Fever, - - - - - - - 657
BIBLIOGRAPHICAL NOTICES: A Treatise on the Diseases of the Heart and Great Vessels, and on the Affections which may be mistaken for them. By J. Hope, M.D., F.R.S. With Notes, and a Detail of Recent Experiments. By C. W. Pennock, M.D., &c., - - - - - - - 664
The London Dissector, or Guide to Anatomy, for the use of Students. By Edward J. Chaisty, M.D., - 665
Report of the Joint Special Committee, on the Subject of the Effects of Lead Pipes upon Well-water in the city of Lowell. By Dr. Samuel L. Dana, - - - - - 665
EDITORIAL: Pathological Society of Philadelphia, - - - - - 665
ANALECTA: Mettauer on the use of the Unripe Persimmon, or Diospyros Virginiana, as a Therapeutic Agent, - - - - - 665
Solan on the Pommade of Oxide of Zinc in Eczema, Impetigo, and Ecthyma, - - - - - - - 668
Warren on the Excision of the Tonsils, - - - - - 668
Channing's Notes on Anæmia, - - - - - - 669

